# HS and Inflammation: A Potential Playground for the Sulfs?

**DOI:** 10.3389/fimmu.2020.00570

**Published:** 2020-04-03

**Authors:** Rana El Masri, Yoann Crétinon, Evelyne Gout, Romain R. Vivès

**Affiliations:** Université Grenoble Alpes, CNRS, CEA, Institut de Biologie Structurale (IBS), Grenoble, France

**Keywords:** heparan sulfate (HS), inflammation, glycosaminoglycan/protein interactions, sulfatase, chemokine, leukocyte migration

## Abstract

Heparan sulfate (HS) is a complex polysaccharide abundantly found in extracellular matrices and cell surfaces. HS participates in major cellular processes, through its ability to bind and modulate a wide array of signaling proteins. HS/ligand interactions involve saccharide domains of specific sulfation pattern. Assembly of such domains is orchestrated by a complex biosynthesis machinery and their structure is further regulated at the cell surface by post-synthetic modifying enzymes. Amongst them, extracellular sulfatases of the Sulf family catalyze the selective removal of 6-*O*-sulfate groups, which participate in the binding of many proteins. As such, increasing interest arose on the regulation of HS biological properties by the Sulfs. However, studies of the Sulfs have so far been essentially restricted to the fields of development and tumor progression. The aim of this review is to survey recent data of the literature on the still poorly documented role of the Sulfs during inflammation, and to widen the perspectives for the study of this intriguing regulatory mechanism toward new physiopathological processes.

## Introduction

Heparan sulfate proteoglycans (HSPGs) are major components of the cell surface, extracellular matrix (ECM) and basement membrane in most animal cells. They are composed of a protein core, onto which are covalently attached complex, anionic Heparan Sulfate (HS) chains of the glycosaminoglycan (GAG) polysaccharide family. Through the ability of their HS chains to bind, modulate and control bioavailability of a multitude of protein ligands, HSPGs are involved in a plethora of biological processes, including cell adhesion, migration, proliferation and differentiation, embryo development, inflammation, control of angiogenesis, blood coagulation, tumor growth, and metastasis (1–3). Structurally, a HS chain is characterized by the linear repetition of a disaccharide unit, composed of alternating N-acetyl glucosamine (GlcNAc) and glucuronic acid (GlcA). During biosynthesis, the polymer undergoes a series of modifications, which include the *N*-deacetylation/*N*-sulfation of glucosamine to form N-sulfo glucosamine (GlcNS), the C5 epimerization of GlcA into iduronic acid (IdoA), and *O*-sulfations at positions C2 of IdoA and C6 (more rarely C3) of glucosamine residues. Tight regulation of these modification steps leads to the generation of specialized saccharide regions termed S-domains, exhibiting both remarkable structural diversity and high sulfation content. These S-domains are involved in the recognition and binding of most HS ligands (4–6). HS is structurally closely related to heparin, although the latter displays a much higher sulfation content. Therefore, heparin has been widely used as a surrogate of HS S-domains for protein interaction studies.

## HS in Inflammation

Inflammation is a complex, multi-step process leading to the rapid recruitment of leukocytes from the blood to the inflammation site. Briefly, emission of an inflammatory signal triggers the secretion of cytokines and chemokines that diffuse throughout the tissue and activate leukocytes and vascular endothelial cells. Cell activation leads to the adhesion and rolling of leukocytes on the endothelium toward the inflammatory site. Leukocytes will then cross the endothelium to the basement membrane, and migrate toward the inflamed tissue to initiate immune responses. Through its large protein binding properties, HS participates in various steps of inflammation [for review, see (7, 8)].

Inflammation is first initiated by the production, upon endogenous or exogenous signals, of inflammatory cytokines. Amongst these, chemokines are a family of small proteins involved in many biological processes such as development, inflammation and immunosurveillance (9). Chemokines induce the activation of the endothelium and the migration of leukocytes from blood toward inflammatory sites. To elicit their functions, they bind to their primary G-coupled receptors that trigger downstream signaling. In addition, all chemokines bind to cell surface and ECM HS. The structural basis of these interactions has been intensively studied and is now well documented (10–12).

Functionally, HS does not seem to be essential for chemokine signaling *in vitro*. However, *in vivo* studies showed that chemokines unable to bind to HS failed to recruit leukocytes (13), and that HS modulated chemokine activity through different mechanisms [for review, see (12, 14)]. HS first regulates chemokine diffusion and sequestration. In some instance, the capture of the chemokine/cytokine by HS prevents its release and thus its activity (15). However, by regulating chemokine diffusion, HS participates in the formation and stabilization of chemotactic gradients providing directional cues for migrating leukocytes. In support to this, *in vivo* inhibition of CXCL12/HS interaction using sulfated polysaccharide tilted the chemokine distribution from bone marrow toward the plasma, thereby causing the release of hematopoietic progenitor cells in the blood circulation (16). HS also mediates chemokine transcytosis across the endothelial cell wall (17, 18), and protects chemokines/cytokines from enzymatic degradation and inactivation (19–21). Finally, HS may further modulate chemokine activity by inducing chemokine oligomerization [for review, see (11)], which has been shown to be functionally relevant *in vivo* (13). In that context, an original HS-dependent cooperative mechanism driving CCL5 dimerization has been characterized (22).

Activation by pro-inflammatory chemokines and cytokines induces the expression of endothelial C-type lectins E- and P-selectins. These bind a variety of glycosylated ligands present on the leukocyte cell surface to initiate adhesion and rolling of leukocytes on the endothelium. Recruitment is further promoted through additional interactions, involving L-selectin constitutively expressed on leukocytes with leukocyte and endothelial ligands (23, 24). HS may participate to this process, as studies reported binding to L-selectin (24, 25), P-selectin (26), and E-selectin (27, 28). However, it should be noted that the physiological relevance of these interactions remains to be clarified. Studies showed that the removal of cell surface HS with heparinases reduced L-selectin dependent binding of monocytes, and leukocyte rolling on endothelial cells (29, 30). During acute inflammation, HS was shown to support L-selectin dependent rolling of neutrophils on lung microvasculature (31). On the contrary, lymphocyte rolling on high endothelial venules (HEV) exclusively relied on interactions of L-selectin with ligands bearing sialyl Lewis X (sLe^x^) glycosylation motifs, suggesting no involvement of HS in L-selectin-dependent lymphocyte homing (32). One could speculate that these discrepancies could be due to the presence of distinct HS structures and/or sLe^x^ ligands on these different cell types.

Leukocyte rolling is then arrested through increased integrin-mediated cell-cell adhesion. In a recent study, it has been proposed that endothelial cell surface HS could participate indirectly to this process, by capturing and presenting CCL21, which would in turn activate integrin LFA-1 on rolling lymphocytes (32). Following arrest, leukocytes get access to inflamed tissues through extravasation across the endothelial cell wall. They then reach the basement membrane that comprises numerous interacting molecules and a variety of HSPGs, including perlecan, agrin and XVIII collagen, which may further modulate the extravasation process. These HSPGs can bind many chemokines, cytokines and growth factors that are critical for leukocyte migration, and contribute to the formation of chemokine gradients. On the contrary, they can act as a physical barrier hindering leukocyte migration.

Finally, studies have also suggested a role of HS in the phagocytosis process (33, 34). A proposed mechanism is that newly-exposed HS binding sites at the surface of apoptotic cells could facilitate their recognition, uptake and clearance by macrophages (35). However, *in vivo* data are still needed to clarify this process.

Most of these inflammatory steps are generally accompanied by changes in the expression of their cell-surface HPSGs and HS structure. Many studies have reported an upregulation of cell-surface HSPG Syndecan-1 upon endothelial cell activation by pro-inflammatory cytokines [reviewed in (25)]. Differentiation of monocytes into macrophages leads to high expression of Syndecan-1, -2 and -4, whereas macrophage activation by Interleukin (IL)-1 results in the overexpression of Syndecan-2 only (36). Furthermore, the activation of T cells induces the expression of Syndecans and Glypicans, while the differentiation of B cells into plasma cells specifically triggers Syndecan-1 expression (37, 38). Depending on the inflammatory *stimuli*, HS length, structure and sulfation profiles may also be affected. For instance, the size and 6-*O*-sulfation patterns of HS are altered in primary human endothelial cells upon treatment with tumor necrosis factor (TNFα) or IL-1 (39). Pro-inflammatory effectors have been shown to modulate the expression of NDSTs (40–42). In particular induction of NDST1 led to the production of highly sulfated HS that increased the sequestration of CCL5, thereby promoting leukocyte extravasation (42). Vascular lesions in mice have also been associated with a significant increase of NDST1 expression (43). In line with this, inactivation of NDSTs in endothelial cells led to impaired rolling and infiltration of neutrophils and macrophages into inflammatory sites (31, 44). In contrast, restricted inactivation of NDSTs to leukocytes had no effect on leukocyte infiltration (31). Changes in HS *O*-sulfotransferases (OSTs) expression have also been reported, during macrophage M1/M2 polarization (45), or the development of renal fibrosis (46). Interestingly, the stimulation of human monocytes by LPS or TNFα upregulates only one out of the seven 3OST isoform: 3OST3b, thus highlighting the fine tuning of sulfotransferase expression pattern during inflammation (47). Finally, the silencing of 2OST in mouse endothelial cells during acute inflammation resulted in enhanced HS 6-*O*- and *N*-sulfation, leading to increased neutrophil infiltration (48).

## Post-Editing Mechanisms Regulating HS Distribution, Structure and Function

Although HS expression and structure is primarily controlled during polysaccharide biosynthesis, increasing evidence have also highlighted the importance of the additional regulatory step provided by post-editing enzymes, including Heparanase, sheddases, and sulfatases of the Sulfs family.

In inflammatory processes, post-synthesis regulation of HS plays a significant role. Heparanase is an endo-β-D-glucuronidase targeting [GlcA-GlcNS] linkages within HS. Heparanase cleaves long HS chains from ECM and cell-surface HSPGs into shorter fragments of 10–20 sugar units. This results in the release of sequestered HS bound ligands, such as growth factors, chemokines and morphogens, which can then induce angiogenesis, cell proliferation and motility (49). Consequently, Heparanase has been associated with various pathologies, including cancer, inflammation, thrombosis, atherosclerosis, fibrosis, diabetes, and kidney disease (50). During inflammation, Heparanase plays multiple roles. It favors neutrophil adhesion onto the endothelium, by degrading endothelial cell-surface HS and unmasking membrane adhesion molecules (51). It also facilitates leukocyte extravasation, by degrading basement membrane HSPGs (52). More recently, Heparanase has been shown to enhance T-cell activity (53). Furthermore, intracellular heparanase upregulated the transcription of genes involved in T-cell differentiation (54). Finally and noteworthy, Heparanase expression is also markedly increased during neuroinflammation (55).

Shedding of HSPGs takes place upon activation of metalloproteinases, plasmins and elatases by inflammatory cytokines (56–59). These proteases target the core protein of HSPGs like Syndecans, thus releasing soluble HS-peptide conjugates. Shedding regulates the amount of HSPGs found at the cell surface or in the ECM and facilitate the release of sequestered chemokines, which contributes to the resolution of neutrophilic inflammation (58, 60). In combination with Heparanase, shedding also facilitate leukocyte migration by altering the architecture of the ECM and basement membranes (52).

In addition, soluble released HS fragments/conjugates (produced by sheddases or Heparanase) induce the secretion by macrophages and splenocytes of pro-inflammatory cytokines through activation of the NF-κB signaling pathway (61–63). Soluble HS fragments also activate neutrophils and promote an immune response via toll like receptor-4 (64, 65). They can also trigger the maturation of dendritic cells, leading to their migration toward lymphoid organs to elicit primary immune responses (66). In line with this, it was shown that an exogenous administration of HS or elastase resulted in a systemic inflammatory response syndrome (SIRS)-like reaction (67).

## The Sulfs: Post-Synthetic Regulators of HS Structure and Function

**Figure 1:**
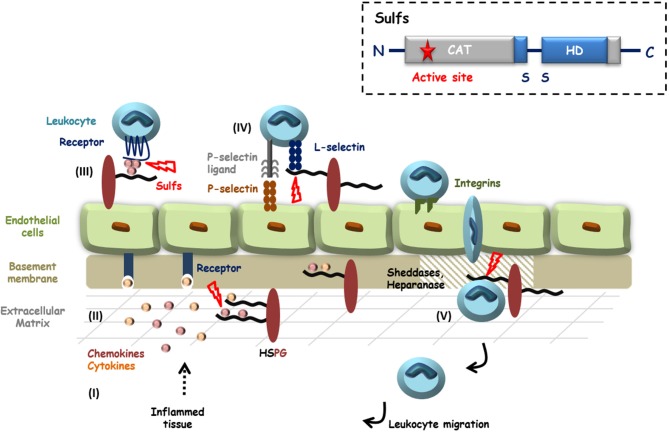
HS in inflammation and potential roles of the Sulfs. Inflammatory *stimuli* induce the secretion of cytokines and chemokines (I) that activate endothelial cells and blood circulating leukocytes. HS controls the diffusion of these pro-inflammatory proteins, their oligomerization and the establishment of chemotactic gradients (II and III). Activated Leukocytes then adhere and roll over endothelial cells, through interactions of E- and P-selectins with their counter ligands. L-selectin tightens cell-contacts by binding to sLe^X^ decorated glycoproteins and HSPGs (IV). After passage through the endothelial layer, efficient migration of leukocytes toward inflammatory sites requires the degradation of basement membrane HSPGs by proteases and Heparanase (V). HS is thus largely involved during inflammation and HS 6-*O*-sulfation is critical for most of these interactions. Although still poorly investigated, Sulfs could therefore play different roles during inflammation. In the figure, steps during which Sulfs could be implicated are highlighted with red bolts. Inset: schematic representation of Sulf structural organization.

Sulfs are extracellular endosulfatases that catalyze the 6-*O*-desulfation of HS. Sulf-1 was first discovered in Quail (68), then orthologs were later identified in mouse, rat, chick, *C. elegans*, zebrafish, and human, as well as a second related enzyme, Sulf-2 (69). In human, HSulf-1 and HSulf-2 (encoded by two distinct genes) feature a common structural organization (69). Maturation of the sulfs involves furin-type processing of pro-enzyme proteins, yielding two sub-units linked by one or more disulfide bonds (see Figure 1 inset). The N-terminal regions features the enzyme catalytic (CAT) domain, which shows strong homology with most eukaryotic sulfatases. The CAT domain comprises the enzyme active site, including the two conserved sulfatase signature amino acid sequences as well as the cysteine modified, catalytic FGly residue. The second functional domain of the Sulfs is the highly charged basic domain (HD), which spans over both N-terminal and C-terminal sub-units. The HD domain is a unique feature of the Sulfs, as it shares no homology with any other known protein. It is responsible for high affinity binding to HS substrate and is therefore required for the enzyme endo-6-*O*-sulfatase activity (70–73). In addition, the HD domain has been shown to mediate the capture of Sulfs on cell-surface HS thereby modulating enzyme diffusion (71). Finally, Sulf C-terminal region shows homology to Glucosamine-6-sulfatase (G6S) and *Arabidopsis thaliana* GlcNAc transferase, suggesting a role of this domain in the recognition of glucosamine motifs (69).

Sulfs catalyze the 6-*O*-desulfation of HS with a strong preference for [Glc/IdoA(2S)-GlcNS(6S)] trisulfated disaccharides that are essentially found within S-domains. Although abundant in heparin, this disaccharide motif is relatively scarce in HS. Sulf-induced modification of HS structure is therefore structurally subtle, but with great functional consequences, as 6-*O*-sulfation pattern of HS S-domains is critical for the binding of many signaling proteins (74). Sulfs are therefore implicated in a number of physiological and pathological processes, including development, tissue repair, neurodegenerative disease and cancer [for reviews, see (75–77)].

## Discussion: Potential Roles of the Sulfs During Inflammation

Over the recent years, Sulfs have emerged as critical regulators of HS functions, with well documented roles during development and cancer. However and despite growing evidence, Sulfs have remained largely unstudied in the context of inflammation (see Figure 1).

It is first well established that HS 6-*O*-sulfation is a major structural determinant for the interaction with many chemokines, including CXCL12 (78–80), CXCL8 (81, 82), CXCL4 (83, 84) and CCL5 (85). For the latter, the resolution of the CCL5 crystal structure in complex with heparin provided further evidence of the contribution of 6-*O*-S group in the interaction (85). Furthermore, HS mimetic glycopolymers featuring [IdoA(2S), GlcNS(6S)] disaccharides efficiently inhibited CCL5/heparin interaction (86). In this context, it is therefore most likely that Sulfs would significantly affect HS binding properties toward many of these chemokines, with consequences on chemokine oligomerization, storage, bioavailability and diffusion. Interestingly, a recent study investigating chemokine inhibition using synthetic sulfated [IdoA(2S), GlcNS(±6S)]_6_ dodecasaccharides showed that the presence of a 6-*O*-sulfate group on the non-reducing end residue switched saccharide binding properties from CXCL8 toward CXCL12 (87). In light of this, it was also recently reported that HSulfs catalyzed the 6-*O*-desulfation of HS following a processive and orientated mechanism, starting from S-domain non-reducing end (88). Together, these data thus suggest that Sulfs could tune HS binding selectivity for chemokines. Despite these findings and most surprisingly, the effect of Sulfs on HS/chemokine interaction has so far only been investigated in one study, which showed the inhibition of CXCL12-α binding to heparin by the Sulfs in an *in vitro* immunoassay (79).

Studies have reported that the 6-*O*-sulfation of endothelial HS was critical for leukocyte rolling (89), and that 6-*O*-sulfation of heparin was necessary to block L-selectin mediated leukocyte adhesion (80, 90). Sulfs could thus be involved in the control of leukocyte adhesion and migration on the activated endothelium. Vascular glycocalyx and especially endothelial HS undergo significant degradation during inflammation (91). A role of the Sulfs in these mechanisms could thus be postulated, as suggested by a first study on post-septic mice. Sepsis is associated with a hyper-inflammatory process, followed by a delayed period of immunosuppression called compensatory anti-inflammatory response syndrome (CARS), which can lead to secondary infections. This study showed that HS of lung endothelial glycocalyx displayed higher 6-*O*-sulfation content after septic injury, which was due to a downregulation of Sulf-1. Interestingly, the post-septic loss of Sulf-1 was necessary for CARS to occur, as the administration of exogenous recombinant Sulf-1 intravenously reversed the immunosuppression phenotype (92). In the same context, Sulfs could also participate in the degradation of the basement membrane, along with Heparanase and proteases to facilitate leukocyte migration toward inflammatory sites. Although there is no evidence of such an involvement yet, a study preceding the discovery of the Sulfs showed that activation of endothelial cells with pro-inflammatory cytokines led to the detection of a sulfatase activity, which was required for the degradation of the basement membrane (52).

Changes in the expression of the Sulfs upon inflammatory conditions have been reported. An *in vivo* study on renal allograft biopsies showed that Sulf-1 expression was repressed in inflammatory conditions (93). In contrast, HSulf-1 was up-regulated in human fibroblasts upon TNF-α treatment (94). Likewise, TGF-β1 induced the expression of Sulf-2 in renal epithelial cells (95), and of both Sulf-1 and Sulf-2 in lung fibroblasts (96). In line with this, Sulf-2 was overexpressed in idiopathic pulmonary fibrosis (97) and would act as a regulator, in a negative feedback loop, of TGF-β1 signaling in type 2 alveolar epithelial cells (96, 97). Furthermore, it was shown that Sulf-2 expression in type II alveolar epithelial cells played a protective role in epithelial lung injury, inflammation and mortality (98). Surprisingly, Sulf-2 overexpression in human hepatocellular carcinoma cells promoted TGF-β1 signaling (99). Altogether, these data clearly underline the complex interplay between Sulf activities and TGF-β1 signaling.

One still intriguing and yet poorly understood issue about the Sulfs is that the two human forms, HSulf-1 and HSulf-2, feature very similar enzyme activities *in vitro*, but show clear functional discrepancies *in vivo*. As such, a study on Sulf KO mice indicated that both forms exhibit redundant or overlapping functions during development (100, 101), while accumulating data describe opposite activities in cancer (75, 76). Furthermore, it has been reported that alternative splicing of *Sulf1/Sulf2* genes generated functionally active variants. In Quail, a QSulf-1 variant (QSulf-1B) encoding for a shorter protein form exhibited opposite activities to full length QSulf-1, QSulf-1B inhibiting Wnt signaling and promoting angiogenesis (102). Mammalian variants of Sulf1 and Sulf2 were later identified in tumors (103, 104). The occurrence and biological significance of Sulf variants are still poorly understood. However, some of these variants have been shown to regulate growth factor signaling pathways and to display anti-oncogenic and anti-angiogenic properties (105). Noteworthy, the expression of Sulf-1 and Sulf-2 variants was also reported in the context of inflammation (105). Studying the spatial and temporal expression and activity of the two Sulf forms as well as their respective splice variants may therefore be critical to fully understand the role of these enzymes in a given biological process such as inflammation.

Finally, another interesting observation arose from our recent article reporting the expression and purification of recombinant HSulf-2 (73). In this work, we showed that the protein HD domain was very sensitive to proteolytic digestion. This observation is in agreement with secondary structure predictions based on amino-acid sequence, which suggests the presence of large unstructured regions within the HD domain. Most surprisingly, we found that these degraded forms of HSulf-2 displayed endosulfatase activity. This is in agreement with a previous study, which demonstrated that deletion of the inner region of HSulf-1 HD domain did not abrogate enzyme activity (71). However, partial degradation of the HD domain may significantly affect HS substrate selectivity and/or the HD-dependent immobilization of Sulfs on cell-surface HS (71). It could thus be hypothesized that, under inflammatory conditions, protease-driven processing of the HD could affect both Sulf biological activity and diffusion throughout tissues.

In conclusion, the structural and functional regulation of HS by the Sulfs is undeniably a significant topic of interest, which merits further investigation in the context of inflammation. Evidence of Sulf involvement in inflammatory processes and in the modulation of pro-inflammatory effectors are slowly arising, but progress in the field has been hampered by the complexity of these enzymes, which markedly distinguishes them from other eukaryotic sulfatases. Nevertheless, recent access to purified recombinant protein solved a critical technical bottleneck, which should boost progress in their study. In this context, we reported for the first time the intravenous injection of recombinant enzyme *in vivo* to analyze an inflammatory process (92), and we anticipate that this study will pave the way to further investigations in this field.

## Author Contributions

All authors listed have made a substantial, direct and intellectual contribution to the work, and approved it for publication.

### Conflict of Interest

The authors declare that the research was conducted in the absence of any commercial or financial relationships that could be construed as a potential conflict of interest.
